# C3aR signaling and gliosis in response to neurodevelopmental damage in the cerebellum

**DOI:** 10.1186/s12974-019-1530-4

**Published:** 2019-07-04

**Authors:** Kevin G. Young, Keqin Yan, David J. Picketts

**Affiliations:** 1 Regenerative Medicine Program, Ottawa Hospital Research Institute, Ottawa, ON K1H 8L6 Canada; 2 Present address: Therapeutic Products Directorate, Health Canada, 1600 Scott St, Ottawa, ON K1A 0K9 Canada; 3 Department of Cellular and Molecular Medicine, University of Ottawa, Ottawa, ON K1H 8M5 Canada; 4 Department of Biochemistry, Microbiology, and Immunology, University of Ottawa, Ottawa, ON K1H 8M5 Canada

**Keywords:** Bergmann glia, C3aR1, C3 complement, Macrophage, Microglia, MerTK, Efferocytosis, Gliosis, Snf2h, VGF

## Abstract

**Background:**

Conditional ablation of the *Smarca5* gene in mice severely impairs the postnatal growth of the cerebellum and causes an ataxic phenotype. Comparative gene expression studies indicated that complement-related proteins were upregulated in the cerebellum of *Smarca5* mutant mice. Complement proteins play critical roles within innate immune signaling pathways and, in the brain, are produced by glial cells under both normal and pathological conditions. The C3 complement protein-derived signaling peptide, C3a, has been implicated in contributing to both tissue damage and repair in conditions such as multiple sclerosis and stroke. Here, we investigated whether C3a receptor (C3aR) signaling promoted damage or repair in the developing cerebellum of *Smarca5* mutant mice.

**Methods:**

Brain and cerebellum lysates from single *Smarca5* conditional knockout (*Smarca5* cKO) mice, *C3aR1* KO mice, or double mutant mice were used for qRT-PCR and immunoblotting to assess the contribution of C3aR to the *Smarca5* cKO brain pathology. Immunohistochemistry was used to characterize alterations to astroglia and phagocyte cells in the developing cerebellum of each of the genotypes.

**Results:**

C3aR signaling was observed to limit gliosis and promote granule neuron survival during postnatal cerebellar development. In *Smarca5* cKO mice, disorganized astroglia with increased GFAP expression develops concurrently with cerebellar granule neuron loss and phagocyte invasion over the first 10 days following birth. Potential ligand precursors of C3aR—VGF and C3—were found to have upregulated expression and/or altered processing during this time. Phagocytes (microglia and macrophages) in both the control and *Smarca5* mutant mice were the only cells observed to express C3aR. Loss of C3aR in the *Smarca5* cKO cerebellum resulted in increased numbers of apoptotic cells and early phagocyte invasion into the external granule cell layer, as well as an exacerbated disorganization of the Bergmann glia. The loss of C3aR expression also attenuated an increase in the expression of the efferocytosis-related protein, MerTK, whose transcript was upregulated ~ 2.5-fold in the *Smarca5* mutant cerebellum at P10.

**Conclusions:**

This data indicates that C3aR can play an important role in limiting astrogliosis and regulating phagocyte phenotypes following developmental cell loss in the brain.

**Electronic supplementary material:**

The online version of this article (10.1186/s12974-019-1530-4) contains supplementary material, which is available to authorized users.

## Background

During the early development of the cerebellum, the *Smarca5* gene is expressed prominently within the external granule cell layer (EGL) [1]. Mouse *Smarca5* expression in cerebellar granule cells peaks within the first 10 days after birth [2], and the Snf2h (sucrose nonfermenting protein 2 homolog) chromatin remodeling protein produced by this gene correspondingly peaks in abundance in the cerebellum within this time period [3]. The loss of *Smarca5* expression through targeted deletion within the mouse nervous system results in a large loss of cerebellar granule neurons and the formation of a small, abnormal cerebellum [3].

We have previously demonstrated a beneficial role for a neuropeptide, VGF, in ameliorating the phenotype of *Smarca5* mutant mice [4]. This was observed in mice post-weaning, after having experienced a significant loss of neurons. This effect was mediated, at least in part, by a promotion of new myelination. Interestingly, the receptors through which VGF signal are complement protein receptors, C3aR and gC1qR [5, 6]. These receptors bind to a C-terminal VGF peptide, TLQP-21, which may mediate part of the benefit to the *Smarca5* mutant brains [4]. Additionally, RNA-seq expression data from this study had indicated that complement protein transcripts are upregulated in the *Smarca5* mutant cerebellum. This has led us to examine the impact of complement-related signaling on the phenotype of the *Smarca5* mutant mice.

Complement proteins, key regulators of innate immunity, can either worsen or improve the central nervous system (CNS) pathologies. For instance, C3 complement protein can promote damage during the acute stage of a stroke and also promote improved long-term repair in the weeks following the stroke [7, 8]. Similarly, complement signaling can exacerbate experimental autoimmune encephalomyelitis, a model for multiple sclerosis [9], and may contribute to MS disease progression [10]. However, beneficial roles such as the promotion of re-myelination by complement signaling and removal of irreversibly damaged cellular material in MS have also been demonstrated [11]. The C3a receptor (C3aR), a key component of complement protein signaling, is an important central mediator of these effects.

C3a signaling has historically been viewed as promoting inflammation [12], and C3aR has been viewed as a marker of neuroinflammation [13]. C3aR expression is upregulated in both stroke and multiple sclerosis, with its expression having been demonstrated to be increased on glial cells in the brain [13, 14]. As well, an influx of C3aR-expressing immune cells post-stroke is associated with increased damage, and inhibitors of C3aR have been proposed for use in stroke therapy [15]. On the other hand, C3a treatment provided during the post-acute phase of stroke can promote regeneration and recovery [8]. Analysis of C3aR knockout mice has also demonstrated anti-inflammatory roles for this receptor [16]. Thus, the role of C3aR in promoting or limiting neuroinflammation, and in exacerbating or remediating damage within the CNS, is context dependent.

In the current study, we demonstrate that C3aR signaling in young mice has a role in limiting astroglial inflammation and structural disorganization and in regulating the phenotype of phagocytic cells following developmental brain damage. In *Smarca5* cKO mice, phagocyte cells invade the EGL of postnatal day 1 (P1) to P10 mice and can be observed phagocytosing apoptotic bodies within this layer. Concurrent with this, Bergmann glia, specialized astroglial cells, display abnormal process structuring and fail to position themselves properly within the cerebellum. This phenotype is worsened by the loss of C3aR. Within the phagocyte population, the expression of a key receptor involved in the clearance of apoptotic cells, MerTK, is dysregulated in the absence of C3aR. These results highlight a role for C3aR in enabling the clearance of dead cells and limiting glial inflammation and further disorganization in the developing *Smarca5* deficient cerebellum.

## Methods

### Mice

The generation of mice deficient for Snf2h in the brain made use of a mouse line with a floxed exon 5 allele of the *Smarca5* gene. As described previously [3], these mice were bred to *nestin-cre*^+/−^ mice which were additionally heterozygous for a *Smarca5* null allele. The resulting conditional knockout (cKO) mice were therefore *Smarca5*^*fl/−*^*;nestin-cre*^*+/−*^. In contrast to our prior studies using these animals, mice in the current study were outbred onto a mixed background that included C57BL/6 N, FVB/N, and BALB/c. Characterization of the *Smarca5*^*fl/−*^*;nestin-cre*^*+/−*^ animals on the mixed genetic background demonstrated that the phenotype was identical to the mice on the C57BL/6 N background with one exception, they were more robust and did perish at P40. Since the C57BL/6 N *Smarca5*^*fl/−*^*;nestin-cre*^*+/−*^ animals could survive past P40 by providing them with unlimited access to a running wheel at weaning (P21), a cohort of singly housed *Smarca5*^*fl/−*^*;nestin-cre*^*+/−*^ and *Smarca5*^*fl/−*^*;nestin-cre*^*−/−*^ mixed background animals (*n* = 3) were provided with unlimited access to a running wheel beginning at weaning (P21) until sacrifice at P35.

*Smarca5*^*fl/−*^*;nestin-cre*^*+/−*^ animals were made C3aR deficient by breeding in a null allele from a *C3aR1* mutant line maintained on a BALB/c background (C.129S4-*C3ar1*^*tm1Cge*^/J; from The Jackson Laboratory). Thus, *Smarca5* cKO, *C3aR* KO double mutants and control animals were generated by breeding *Smarca5*^*fl/fl*^*;C3aR1*^*−/−*^ mice to either *Smarca5*^*+/−*^*;nestin-cre*^*+/−*^*;C3aR1*^*−/−*^ mice or to *Smarca5*^*+/−*^*;nestin-cre*^*+/−*^*;C3aR*^*+/−*^ mice. Samples used as controls were *Smarca5* wild-type (*Smarca5*^*fl/fl*^, *cre*−) and C3aR heterozygous littermates. To examine the background strain differences of our mice, we sent tail DNA for SNP analysis against C57BL/6 N, FVB/N, and BALB/c reference strains (Taconic) from the following lines: Smarca5^*f/f*^ (C57BL/6) mice, Smarca5 ^*f/f*^ (C57BL/6 N; FVB/N mixed), and the resulting lines used to generate the mice for our experiments, namely *Smarca5*^*+/−*^*;nestin-cre*^*+/−*^*;C3aR*^*+/−*^, and *Smarca5*^*fl/fl*^*;C3aR1*^*−/−*^ mice. The results from this analysis are shown in Additional file 1.

Smarca5; *VGF* floxed mice used in this study have been described elsewhere [17]. *Smarca5*, *VGF* mutants were similarly generated using a nestin-cre driver. *VGF*^*f/f*^*;Smarca5*^*f/−*^*;nestin-cre*^*+/−*^ mice were found to die soon after birth. All mice were housed and bred at the University of Ottawa animal facility. All animal experiments were approved by the University of Ottawa’s Animal Care ethics committee, with the guidelines set out by the Canadian Council on Animal Care.

### mRNA analysis

Total RNA was collected from mouse tissues using mechanical homogenization in Trizol (ThermoFisher Scientific) and subsequent RNA isolation following the company’s recommended protocol. DNaseI (ThermoFisher Scientific) was used to remove any contaminating gDNA. cDNA was generated from the purified RNA using random hexamer oligonucleotides and RevertAid reverse transcriptase (ThermoFisher Scientific). Quantitative expression analysis was performed using oligonucleotide primers (Sigma) specific for *VGF*, *GFAP*, *C3*, *C3aR*, *gC1qR*, *Iba1*, *MerTK*, *scavenger receptor-B1* (*SRB1*), *MFG-E8*, *IL6*, and *TNF* (sequences are provided in Additional file 2). Oligonucleotide primers for *GAPDH* and/or *B-actin* were used to amplify a reference cDNA for normalization of all samples. qPCR was performed on a Stratagene Mx3000P system using a SensiFAST SYBR Lo-ROX kit (Bioline). Relative expression fold changes were calculated using the 2^−ΔΔCt^ method, and ranges were calculated using the standard error of the Ct values being added or subtracted to the ΔΔCt values. Statistical differences were calculated using *t* tests comparing the control and individual mutant groups or between two individual mutant groups where noted. Paired *t* tests were performed using the Ct values that had been read in triplicate for samples from each animal.

RNAseq analysis was performed as previously described [4]. Briefly, following Trizol extraction, mRNA was concentrated with MinElute cleanup kits (Qiagen). Three independent cerebellums from each genotype were pooled for individual samples, and two independent pools per genotype were sequenced. Sequencing was performed using Illumina HiSeq 2000 paired-end technology at McGill University and the Genome Quebec Innovation Center.

### Immunoblotting

Tissues were lysed by mechanical homogenization in a lysis buffer containing 150 mM NaCl, 1% NP40, 0.1% SDS, 50 mM pH 8.0 Tris, 5 mM EDTA, and a protease inhibitor cocktail (Sigma, cOmplete cocktail), then boiled for 5 min. Immediately, samples were quantified using a standard Bradford assay, mixed in Laemmli buffer, and run by SDS-PAGE. The protein was transferred onto nitrocellulose membranes, blocked in TBS containing 5% non-fat dairy milk (NFDM). Membranes were incubated in primary and secondary antibodies diluted in TBS + 0.05% Tween-20 containing 5% NFDM.

**Fig. 1:**
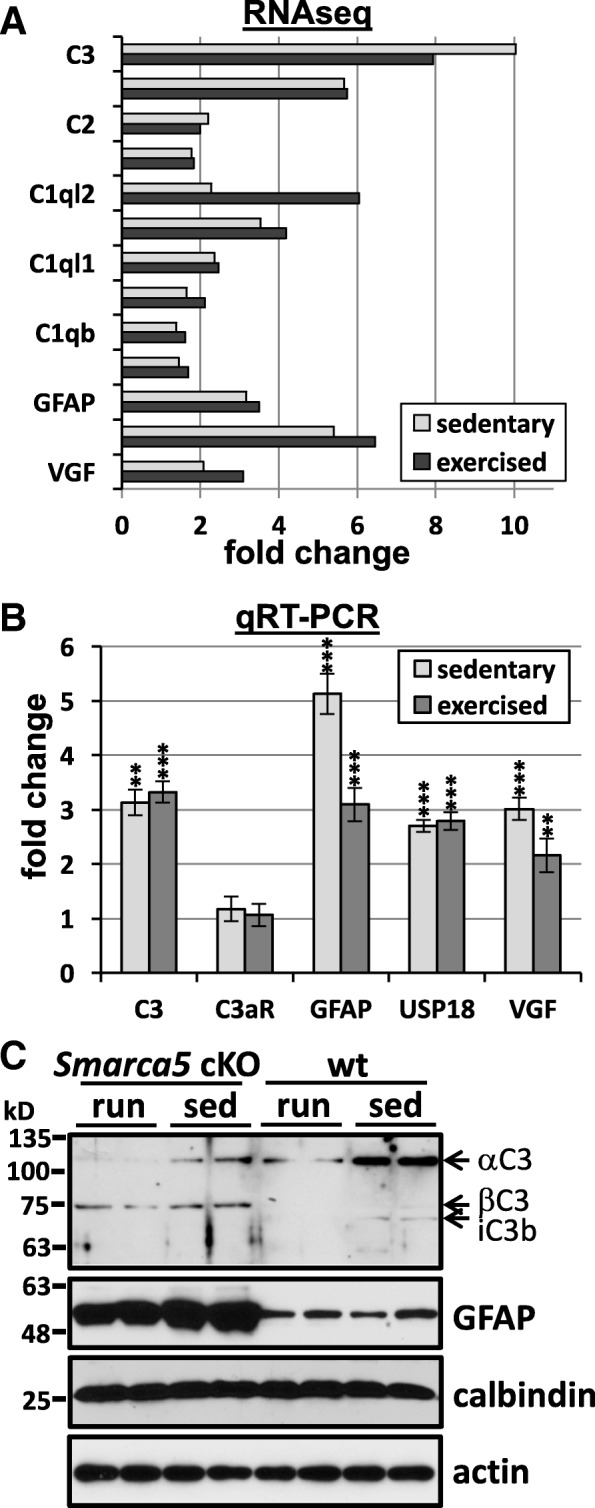
Altered C3 complement protein expression in the *Smarca5* cKO cerebellum of exercised and sedentary mice. Increases in mRNA transcripts coding for complement, complement-related proteins, and inflammation-related proteins in the *Smarca5* cKO cerebellum, as indicated by RNAseq analysis (**a**). Fold changes are shown for the *Smarca5* cKO groups (sedentary or exercised) relative to corresponding wild-type groups. qRT-PCR analysis confirmed the increases in C3, GFAP, USP18, and VGF (**b**), though the magnitudes of these increases varied from the RNAseq data set. Shown are the fold changes in the *Smarca5* cKO cerebellum relative to wild-type littermates (*n* = 3 in each of the four groups of wild-type exercised (run) or sedentary (sed), and mutant exercised or sedentary animals; differences relative to wild-type littermates are noted with **p < 0.005 and ***p < 0.001). No increase was observed for the C3a receptor, C3aR. Protein analysis demonstrated a clear increase in GFAP expression in *Smarca5* cKO cerebellum samples (**c**). C3 protein expression was also altered in the *Smarca5* cKO cerebellum. The C3α chain was less prominent relative to the C3β chain in *Smarca5* cKO cerebellum samples compared to wild-type samples. Blotting results are representative of similar results from four mice/group

For immunoblot quantification, high-resolution scans were analyzed in Image J using Fiji [18]. Briefly, mean gray values and the inverted pixel density were calculated for all bands and corresponding backgrounds using an identical rectangular frame. The net band value (band minus background) was then calculated as a ratio of the net loading control to allow for comparison across lanes. For the GFAP expression plot (Fig. 1), lanes were normalized to the WT sedentary sample to graph the fold increase across samples.

### Immunolabeling of cells and tissue sections

Brains from P1 and P10 mice were immersed in 4% paraformaldehyde overnight at 4 °C, washed in PBS, then immersed in 30% sucrose at 4 °C until saturated. OCT-embedded brains were frozen using liquid nitrogen and stored at − 80 °C. Twelve micrometers of cryostat sections were used for immunolabeling. A standard labeling protocol was used for antibody labeling [19]. Sections were labeled with antibodies against VGF (Santa Cruz, R15 goat polyclonal), GFAP (Santa Cruz, 2A5 mouse monoclonal), Pax6 (Covance, rabbit polyclonal), NeuN (Millipore, mouse monoclonal), cleaved caspase 3 (Cell Signaling Technology, rabbit polyclonal), calbindin (Sigma, CB-955 mouse monoclonal and rabbit polyclonal), BLBP (Abcam, rabbit polyclonal), Iba1 (Wako, rabbit polyclonal and Novus, goat polyclonal), C3aR (Hycult, 14D4 rat monoclonal), gC1qR (Abcam, 60.11 mouse monoclonal), P2RY12 (Cedarlane, rat polyclonal), and MerTK (ThermoFisher Scientific, DS5MMER rat monoclonal). Appropriate secondary antibodies conjugated to Alexa-fluor 488, Alexa-fluor 555, or Alexa-fluor 647 were used to detect the primary antibodies. A 1:5000 dilution of 1 mg/mL Hoechst 33342 (Sigma) was used to label nuclei. TUNEL labeling was performed using digoxigenin (DIG)-labeled nucleotides in a reaction with terminal transferase (Roche). When performing TUNEL labeling, the sections were pre-treated with ethanol/acetic acid (2:1), then processed with a standard TUNEL labeling reaction. Primary antibodies for Pax6 and DIG (Sigma), followed by the appropriate secondary antibodies, were used to label the sections following the TUNEL reaction.

Conventional wide-field fluorescence microscopy was used for most imaging of the sections with × 20 (0.8NA) or × 40 (1.3NA) objective lenses. Where noted, optical sections were acquired using a Zeiss Apotome. Acquisition and post-processing was performed with Axiovision. A minimum of 3 mice/genotype were analyzed for all histological analysis.

## Results

### Smarca5 mutant mice have increased gliosis and altered expression of complement-related proteins

*Smarca5* cKO mice on a C57BL/6 N background were previously characterized as having smaller brains, with the cerebellum being disproportionately affected [3]. Moreover, exercise-induced changes, including an upregulation of the neuropeptide VGF (non-acronym), were able to promote increased survival in these mice [4]. Since VGF is known to bind to the complement protein receptors, C3aR and gC1qR [5, 6], we reasoned that VGF may function through the complement pathway. As such, the published RNAseq data [4] was re-examined for altered expression in mRNA transcripts coding for complement, complement-related proteins, and inflammation-related proteins. Indeed, the C3 complement protein, GFAP (glial fibrillary acidic protein; a marker of astrogliosis [20]), USP18 (ubiquitin specific peptidase 18; a regulator of microglial activation [21]), and VGF were amongst those transcripts observed to be upregulated upon exercise (Fig. 1a). Interestingly, many also showed increased expression in the sedentary animals suggesting that pathway activation precedes exercise and is induced during altered cerebellar development.

More recently, we have utilized *Smarca5* cKO mice and wild-type littermates generated on a mixed strain background (FVB/N; C57BL/6 N; Additional file 1) because they are able to survive beyond P40 without the need for a running wheel. As these animals appear to present with an otherwise identical phenotype (smaller size, hypoplastic cerebellum, abnormal gait [4]), we examined whether VGF and complement pathway activation was maintained. As such, we performed qRT-PCR for the upregulation of several key transcripts in P35 *Smarca5* cKO mice and wild-type littermates that had either been provided a running wheel at the time of weaning (exercised) or left sedentary (as in our prior study [4]). We observed an increase in C3 transcript (irrespective of exercise) in the mutants on the mixed background and an upregulated expression of the other transcripts tested (VGF, GFAP, and USP18) that was consistent with the changes observed previously on the C57BL/6 N strain (Fig. 1b). The receptor for C3- and VGF-derived signaling proteins, C3aR, showed a small increase by RNAseq analysis, though no significant change in its expression was observed by qRT-PCR. Notably, C3 and GFAP both demonstrated ≥ 3-fold increases in transcript expression as measured by qRT-PCR, with a marked reduction in GFAP expression following exercise (Fig. 1b).

Protein analysis confirmed the changes in GFAP expression (Fig. 1c), with the strongest expression being observed in the cerebellum of sedentary *Smarca5* cKO mice and a slight reduction when provided with a running wheel (Additional file 3). Antibody labeling of the C3 protein detects the α and β chains of the full protein, as well as several cleavage products of both chains, which are not entirely characterized within the CNS. The *Smarca5* cKO mice displayed a consistently altered band pattern compared to WT mice that was indicative of active processing of the C3 protein. Running and Snf2h loss enhanced processing of the C3α chain and reduced cleavage of the β chain (Fig. 1c).

**Fig. 2:**
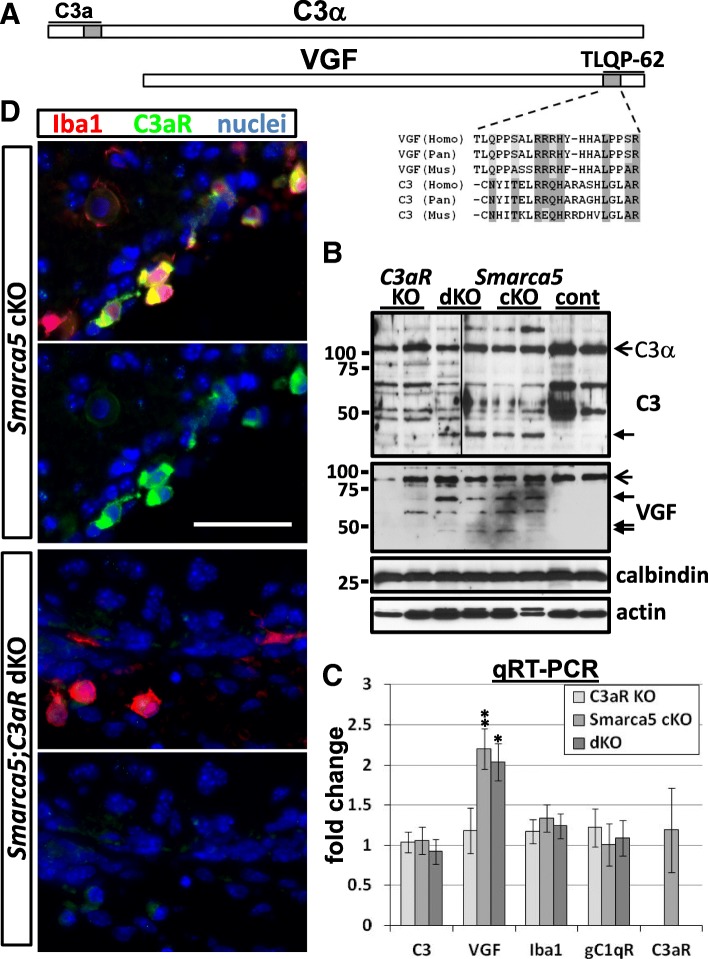
Altered C3 and VGF processing in the P10 *Smarca5* cKO cerebellum. An N-terminal C3α chain sequence (shaded gray) found within the C3a peptide bears structural similarity to a C-terminal VGF sequence (shaded gray) within the TLQP-62 peptide (**a**). Alignment of the human (Homo), chimpanzee (Pan), and mouse (Mus) sequences are shown. C3 and VGF immunoblotting showed additional bands (small closed arrows), presumably corresponding to cleavage products, in *Smarca5* cKO and *Smarca5* cKO, *C3aR* KO double mutant (dKO) cerebellum samples (**b**). Open arrows in **b** indicated full-length C3α and VGF. VGF mRNA was upregulated ~ 2-fold in the P10 *Smarca5* cKO and dKO cerebellum (**c**) (*p < 0.05, **p < 0.005). C3 mRNA expression, however, was unchanged in the mutant cerebellum samples. **d** The common receptor for VGF and C3, C3aR, was detected only in Iba1^+^ macrophages and microglia (Additional file 4). The specificity of the C3aR labeling was demonstrated by the lack of immunolabeling in the dKO cerebellum. Top images in each pair show the merge of Iba1 and C3aR labeling, and bottom images show C3aR labeling alone. Scale bar =50 μm and applies to all images

The C3α-derived C3a peptide and the VGF-derived TLQP-62 peptide share sequence homology (Fig. 2a) and both signal through C3aR [6]. This led us to further investigate a role for C3, VGF, and C3aR signaling in modifying the development of the *Smarca5* cKO cerebellar phenotype. In order to examine whether this signaling may be of importance at a time when the majority of cell loss is occurring due to the primary defect (i.e., loss of Snf2h expression), we performed further experiments in younger (P1 and P10) mice.

We first chose to look at P10, an early stage in cerebellar development when progenitor cells within the external granule cell layer are actively dividing to generate granule neurons. In P10 *Smarca5* cKO cerebellums, processing for both C3 and VGF was altered relative to wild-type littermates (Fig. 2b). Though the C3-immunoreactive bands in the P10 mutant cerebellum differed from the wild-type cerebellum, as was the case in the P35 mutant cerebellum, the sizes of the lower molecular weight bands were different between P10 and P35 (compare Fig. 2b with Fig. 1c). VGF also produced lower molecular weight bands in cerebellum samples from the *Smarca5* cKO mice (Fig. 2b). In trying to assess a role in regulating the *Smarca5* cKO phenotype for C3aR, we bred *Smarca5* cKO mice with *C3aR* KO mutant mice to produce double mutant (dKO) mice. The same lower molecular weight C3 and VGF bands found in the *Smarca5* cKO cerebellums were also found in the dKO mouse cerebellums (Fig. 2b).

Increased expression of VGF transcript, but not C3 or C3aR transcript, was apparent in both the P10 *Smarca5* cKO and dKO cerebellums by qRT-PCR analysis (Fig. 2c). As C3aR was observed to be exclusively a microglial/macrophage receptor in the cerebellum (Fig. 2d), we also examined the expression of Iba1 and gC1qR transcripts (Fig. 2c). Iba1 is a common marker for microglia/macrophage cells, and gC1qR is a microglia/macrophage complement protein receptor that has also been shown to serve as a VGF receptor. No significant changes in expression were observed for Iba1 or gC1qR.

The specific expression of C3aR protein was only observed on border-associated macrophages (BAMs) and microglia by immunofluorescence histology of the cerebellum (Fig. 2d and Additional file 4). BAMs were also the only cell type observed to express both the C3aR and gC1qR complement protein receptors (Additional file 4). Thus, these cells may serve as a target for VGF- and C3-derived peptide signaling in the *Smarca5* cKO mouse cerebellum.

**Fig. 3:**
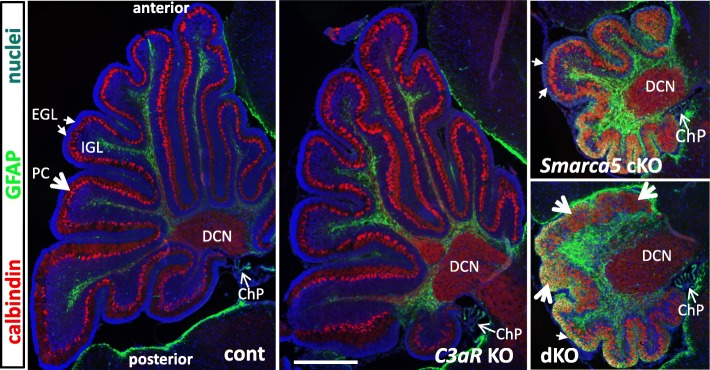
Morphologies of the P10 *Smarca5* cKO and dKO cerebellum. P10 wild-type, *C3aR* KO, *Smarca5* cKO, and dKO cerebellums were labeled to show Purkinje cells (calbindin) and astroglia (GFAP) in sections through the vermis. All sections are oriented with the anterior end towards the top. Large open arrows point to the Purkinje cell layer; small closed arrows point to the external granule cell layer (EGL), and small open arrows point to choroid plexus (ChP). Also labeled is the region of the deep cerebellar nuclei (DCN). Both the *Smarca5* cKO and dKO cerebellums displayed more intense GFAP labelling; this labelling in the dKO was more prominent around the periphery. The dKO also displayed a more pronounced loss of EGL in comparison to the *Smarca5* cKO single mutant. The *C3aR* single mutant did not have any apparent abnormalities in comparison to the wild-type sections. The scale bar in the middle panel = 500 μm, and applies to all images

Loss of C3aR signaling in the *Smarca5* cKO mice had no impact on survival, though the cerebellar phenotype was altered. As with the *Smarca5* cKO mice outbred onto a mixed strain background, dKO mice showed good survival up until at least 100 days of age (80% *Smarca5* cKO survival (*n* = 10) vs. 82% dKO survival (*n* = 11)). The dKO mice were slightly smaller in comparison to *Smarca5* cKO mice, though this was not statistically significant on most days with the number of mice analyzed (Additional file 5). In contrast, *C3aR* KO mice showed a tendency to be slightly larger in comparison to *C3aR* heterozygotes and WT controls. The cerebellum of the dKO mice at P10 was similar to that of the *Smarca5* cKO mice in size, whereas the organization and size of the cerebellum from *C3aR* KO mice was equivalent to WT animals (Fig. 3). In general, the dKO cerebellum exhibited a thinner EGL and increased GFAP labeling around the periphery relative to the *Smarca5* cKO cerebellum. Purkinje cell clustering occurred with the loss of granule neurons in the mutants (see also Additional file 6), and this was more pronounced in the dKO cerebellum. The dKO cerebellums were otherwise similar in appearance to the *Smarca5* cKO cerebellums.

**Fig. 4:**
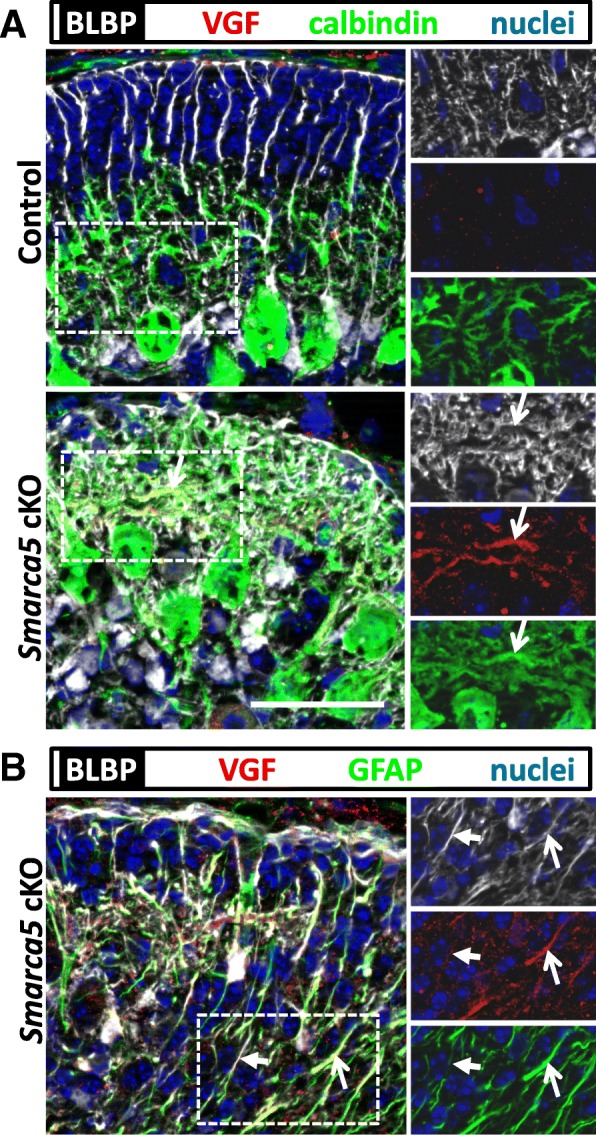
VGF production in P10 *Smarca5* cKO Purkinje cells and Bergmann glia. VGF immunofluorescence labeling generally consisted of diffuse punctae in the cerebellum. Discrete labeling co-localized with calbindin^+^ Purkinje cell dendritic trees (open arrows) of the *Smarca5* cKO mutants (**a**), with similar labeling occurring in the *Smarca5*;*C3aR1* dKO mutants (Additional file 4). Similar labeling was not observed in sections from control animals. Strong VGF labeling was also noted co-localizing with some strongly GFAP^+^ Bergmann glia (open arrows) (**b**). The closed arrows indicate a weakly GFAP^+^ Bergmann glia cell with only faint VGF labeling. BLBP labeling was used to mark Bergmann glia cell bodies and processes in all samples. Right-hand images show the colors separated from the boxed regions of the left-hand images. Scale bar = 50 μm, and applies to all images

To investigate the localization of the upregulated VGF protein, immunolabeling using an antibody directed against the VGF C-terminal end was used. This showed labeling in Purkinje cell dendrites and inflamed Bergmann glia in the *Smarca5* cKO cerebellum (Fig. 4). Similar labeling was also observed in the dKO cerebellum (Additional file 7), but not in control samples (Fig. 4). Bergmann glia inflammation was assessed using GFAP labeling, with a brain lipid binding protein (BLBP) antibody marking both inflamed and non-inflamed Bergmann glia. The VGF labeling was weak or absent in Bergmann glia processes that displayed weaker GFAP labeling (Fig. 4b). In trying to further assess the importance of VGF expression in the *Smarca5* cKO mouse brain, we bred mice that were conditional knockouts in the brain for both *Smarca5* and *VG*F, using the nestin-cre promoter. However, *Smarca5*^f/−^;*VGF*^f/f^;*nestin-cre*^+/−^ mice died soon after birth. *VGF*^f/f^;*nestin-cre*^+/−^ mice showed no early deaths. Thus, VGF expressed within the nervous system is absolutely essential to the survival of the *Smarca5* cKO mice.

### Smarca5 mutant mice deficient for C3aR exhibit increased Bergmann glia disorganization

**Fig. 5:**
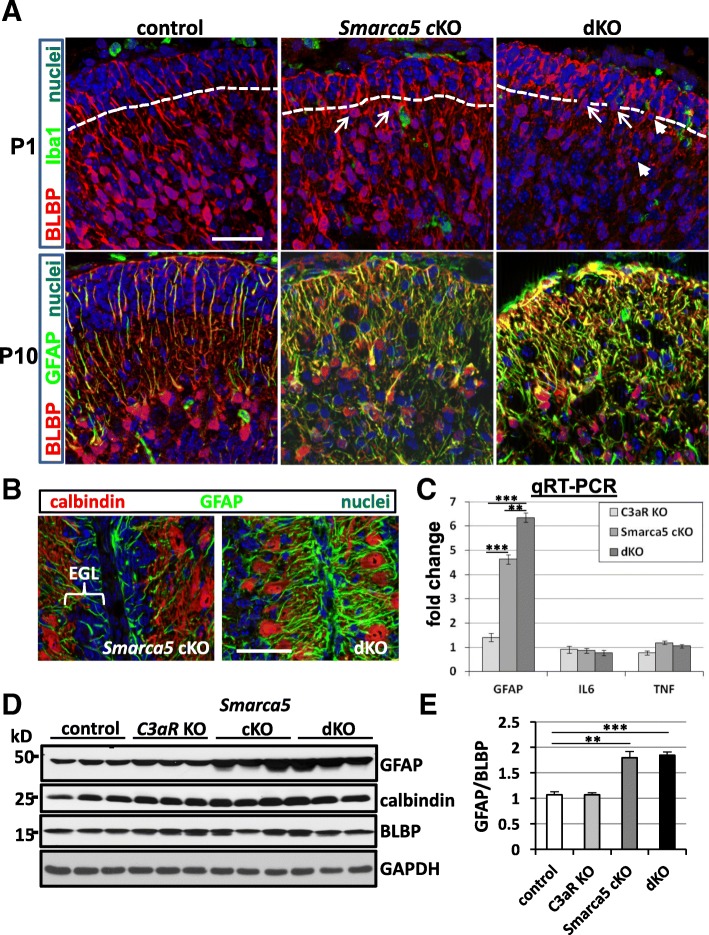
Loss of C3aR in the *Smarca5* cKO cerebellum results in increased Bergmann glia disorganization. At P1, BLBP labeling in mutant cerebellums showed the cell bodies of the Bergmann glia to be mislocalized (**a**), being frequently positioned immediately adjacent to the EGL (arrows; the EGL is indicated with a dashed line). BLBP labeling was further altered in the dKO samples in that it was weak throughout the cerebellum, except within the EGL, where it labeled strongly. Iba1^+^ cells were also labeled to look for any correspondence in Bergmann glia disorganization and phagocyte localization, which was not observed. In P10 samples, the orderly array of Bergmann glia cell bodies within the Purkinje cell layer observed in control samples was mostly absent in both *Smarca5* cKO and dKO samples. Both of these mutants also displayed increased GFAP labeling and aberrant arborization, which was worsened in the dKO sections. Within fissures of the cerebellum (**b**), the nearly absent EGL and strongly GFAP^+^ and disorganized processes of the Bergmann glia in the P10 dKO contrasted with a more normal-appearing *Smarca5* cKO single mutant. Calbindin labeling identified the Purkinje cells. **c** Transcript expression for GFAP was increased in both of the *Smarca5* cKO and dKO cerebellums, being higher in the dKO mutants (*n* = 4 mice/genotype; **p < 0.005, ***p < 0.0001). Transcripts for the inflammatory cytokines IL6 and TNF were not increased (IL6, *n* = 4–5 mice/genotype; TNF, *n* = 5 mice/genotype). **d** Total GFAP protein was increased in both the *Smarca5* cKO and dKO cerebellums relative to cerebellums from controls and *C3aR* KO mice. **e** Quantification of the GFAP/BLBP ratio from the immunoblots shown in D (**p < 0.01; ***p < 0.001). Images in **a** and **b** were reconstructions of optical sections. Scale bar in **a** = 40 μm, and applies to all panels; scale bar in **b** = 40 μm, and applies to both panels

To examine the impact of the loss of signaling through C3aR on the glial phenotypes of the *Smarca5* cKO mutant, we first assessed astroglial morphologies and phenotypes in the *Smarca5* cKO and dKO mutant cerebellums. Both the *Smarca5* cKO and dKO cerebellum displayed disorganization of the Bergmann glia within the periphery of the cerebellum (Fig. 5). This phenotype was greatly exaggerated in the dKO cerebellum. Differences in labeling of BLBP were already evident at P1. BLBP labeling indicated that in both the *Smarca5* cKO and dKO cerebellum. Bergmann glia cell bodies were mislocalized. In the control cerebellum, there was a gap of 20–30 μm between the Bergmann glial cell bodies and the EGL at P1, but in the mutant cerebellums, Bergmann glia cell bodies appeared to be randomly scattered, with some positioned immediately adjacent to the EGL in all sections examined (Fig. 5a). In the dKO, the BLBP signal was further altered, showing strong labeling in the EGL and weak labeling outside of it. Some of the Bergmann glial cells in the dKO also displayed pyknotic nuclei, which was not observed in sections from the other genotypes. At P10, mislocalized Bergmann glia cell bodies and aberrant arborization were increasingly apparent in the mutant cerebellums. In the dKO cerebellum, the mislocalization of the Bergmann glia cell bodies and abnormal arborization resulted in their chaotic appearance, in contrast to the parallel arrangement of the Bergmann glia fibers protruding from cell bodies that were lined up within the developing Purkinje cell layer in the control animals.

Variations in these features were apparent dependent on the lobes observed (Additional file 6), though the peripheral astrogliosis was readily apparent to some degree throughout all of the dKO cerebellums observed (*n* ≥ 5 for each genotype). For the sake of consistency, all histological observations in this study focused on the anterior lobes in the vermis. The exact lobes being analyzed were often difficult to ascertain, however, due to the extensive abnormality in the structuring of the mutant cerebellums. Within the fissures between lobes, the differences in EGL thickness and increased disorganization of GFAP expression in the Bergmann glia of the dKO cerebellums was consistently apparent (Fig. 5b). This also had the effect of leaving little space for normal Purkinje cell arborization.

GFAP transcript expression at P10 was consistent with the increased GFAP immunolabeling. It was increased > 4-fold in the *Smarca5* cKO cerebellum and > 6-fold in the dKO cerebellum (Fig. 5c). Though increases in GFAP are often taken as a sign of inflammation, transcripts for pro-inflammatory cytokines TNF and IL6 were not increased in the mutant mice. Immunoblotting indicated that GFAP protein was increased ~ 2-fold in both of the *Smarca5* cKO cerebellum and dKO cerebellum samples (Fig. 5d, e).

Disruption of normal Bergmann glia structure and increased GFAP labeling may be reflective of a leaky blood-brain-barrier (BBB). To address whether or not the BBB showed signs of compromise, we labeled cerebellum sections with antibodies for the tight junction proteins ZO-1 and claudin5, which are expressed in blood vessel endothelium. As well, we labeled with an antibody against PLVAP, a protein which is downregulated with the maturation of the BBB and expressed only in restricted locations in the postnatal brain. Labeling for ZO-1 and claudin5 was similar between control and *Smarca5* cKO and dKO sections (Additional file 8). On the other hand, PLVAP labeling was absent in the cerebellum sections of each of the genotypes. Combined, this indicates that the BBB was intact in the *Smarca5* cKO and dKO mutant cerebellums.

### Smarca5 cKO mice deficient for C3aR have increased granule neuron apoptosis and altered phagocyte infiltration into the external granule layer

**Fig. 6:**
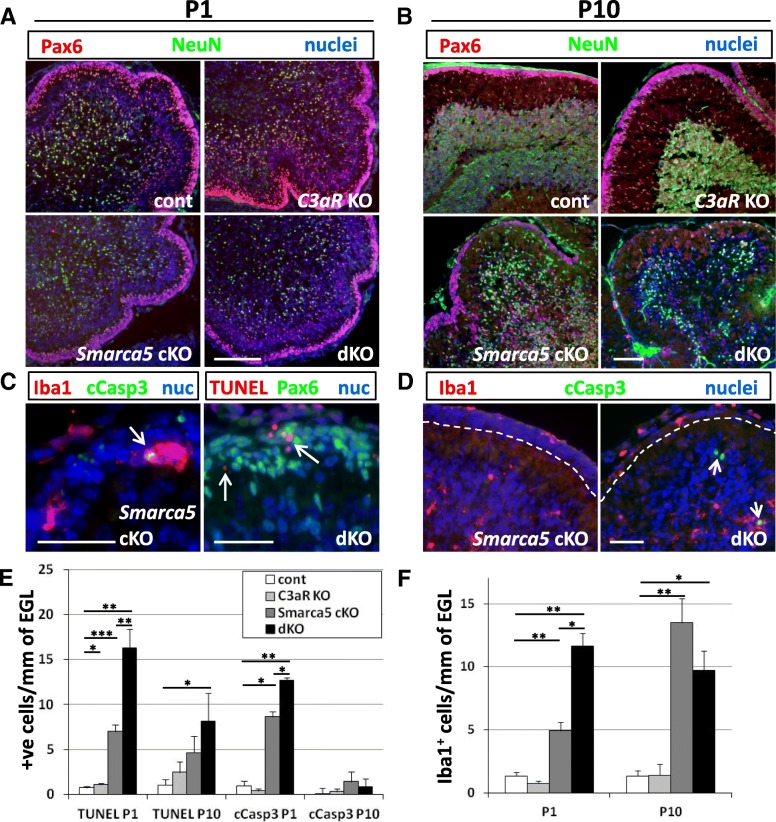
Loss of C3aR in the *Smarca5* cKO cerebellum results in increased apoptosis in the EGL and an early invasion of phagocyte cells. P1 (**a**) and P10 (**b**) cerebellums from control and mutant mice showing granule neuron precursors (Pax6^+^) in the EGL and NeuN^+^ granule neurons. At P10, the loss of Pax6^+^ cells from the EGL was observed in both the *Smarca5* cKO and dKO cerebellum, and NeuN^+^ cells in the IGL were sparse in these mutants in comparison to control and *C3aR* KO cerebellums. Within the EGL of *Smarca5* cKO and dKO mutant mice, cleaved caspase 3^+^ (cCasp3) apoptotic cells and TUNEL^+^ cells (arrows) were found in every section examined (**c**). These cCasp3^+^ cells in the EGL were occasionally observed within Iba1^+^ phagocyte cells (left-hand panel). **d** Iba1^+^ phagocytes were also observed in the EGL of *Smarca5* cKO and dKO mutants at P10, though cCasp3^+^ cells were infrequent at this age. Most cCasp3^+^ cells observed at P10 were in the IGL (arrow, right-hand panel). The number of cCasp3^+^ and TUNEL^+^ cells (**e**) was highest in the dKO mice (*n* = 5 mice/genotype; error bars indicate standard error). However, only P1 dKO cerebellums showed a consistent, statistically significant increase in TUNEL labeling and cCasp3^+^ cells compared to *Smarca5* cKO single mutants. At P10, TUNEL labeling was more variable; only the dKO cerebellums showed a statistically significant increase in TUNEL^+^ cells relative to the non-mutant control group at this age. Both the *Smarca5* cKO and dKO cerebellums had high numbers of phagocytes in the EGL at P10 (**f**), whereas the *Smarca5* cKO mutants had less than half as many as the dKO at P1 (*n* = 4 mice/genotype at each age; error bars indicate the standard error). *p < 0.05, **p < 0.005, ***p < 0.001 in **e**, **f**. Scale bars = 100 μm in all image panels

Despite the eventual granule neuron loss in *Smarca5* cKO mutants, labeling with markers for granule neuron progenitors (Pax6) and mature granule neurons (NeuN) was similar between each of the genotypes at P1 (Fig. 6a). In all genotypes, there was a well-defined EGL with Pax6^+^ progenitors and nascent inner granule cell layer (IGL) with NeuN^+^ cells. However, by P10, when the IGL was densely packed with NeuN^+^ cells in both control and *C3aR* KO cerebellums, the *Smarca5* cKO and dKO mutant cerebellums contained sparsely scattered NeuN^+^ cells (Fig. 6b). At P1, cleaved caspase 3^+^ and TUNEL^+^ cells were readily apparent in the *Smarca5* cKO and dKO mutant EGL (Fig. 6c). The numbers of both cleaved caspase 3^+^ cells and TUNEL^+^ nuclei were highest in the dKO mice (Fig. 6e). By P10, TUNEL labeling, but not cleaved caspase 3 labeling, remained high in these mutants. Most of the cleaved caspase 3^+^ cells were observed only within the IGL at P10 (Fig. 6d). Taken together, this data suggests that increased numbers of dead cells persist in the EGL of the dKO mice throughout the first 10 days after birth and that most of these cells are either late-stage apoptotic cells or necrotic cells that have yet to be removed.

As Iba1^+^ phagocytes were the only cell type to clearly express C3aR, we speculated that differences may exist with this cell population in the absence of C3aR in the presence of abnormal developmental cell death. Iba1^+^ cells were readily apparent in the EGL of the *Smarca5* cKO and dKO cerebellum (Fig. 6d). Iba1^+^ cells were found in higher numbers in the P1 dKO EGL compared to the *Smarca5* cKO EGL and were rarely observed in the EGL of control or *C3aR* KO mice (Fig. 6f). The difference in phagocyte numbers within the EGL of the *Smarca5* cKO and dKO mice disappeared by P10, with high numbers occurring in both relative to controls.

**Fig. 7:**
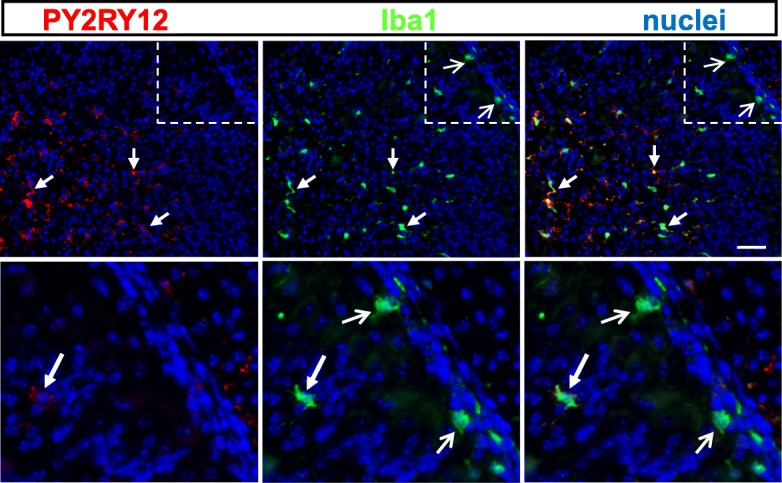
Iba1^+^ cells in the mutant EGL do not express a characteristic microglial marker. Labeling for P2RY12 in the P10 cerebellum of a dKO mutant animal produced co-labeling only with Iba1^+^ cells inside of the EGL (closed arrows). In some of these Iba1^+^ cells, the P2RY12 labeling was relatively limited (closed arrow, bottom panels); however, in the amoeboid shaped Iba1^+^ cells found in association with the EGL (open arrows), no P2RY12 labeling was detectable. The bottom panels are enlargements of the boxed area indicated in the top panels. Scale bar = 100 μm

Co-labeling with an anti-P2RY12 antibody was used to determine whether the Iba1^+^ cells that entered into the EGL of the *Smarca5* cKO and dKO mice expressed a characteristic marker unique to microglia [22]. In the P10 dKO cerebellum, P2RY12 was readily detectable on Iba1^+^ cells within the parenchyma of the cerebellum (Fig. 7). However, of 165 Iba1^+^ cells counted (*n* = 3 animals) within the EGL, very few (12 cells) were co-labeled with P2RY12. A similar finding was also found for the *Smarca5* cKO mice (3 Iba+;P2RY12+ of 108 Iba + cells in the EGL; *n* = 3 animals). This result contrasted sharply with counts from WT or C3aR KO animals, for which there were very few Iba + cells in the EGL (13 and 9, respectively), and all co-labeled with P2RY12 (Additional file 9). This suggests that the Iba1^+^ cells that entered into the EGL of the *Smarca5* cKO and dKO mutant cerebellums were primarily invading macrophages.

### C3aR deficiency blocks an increase in MerTK expression in the Smarca5 mutant cerebellum

**Fig. 8:**
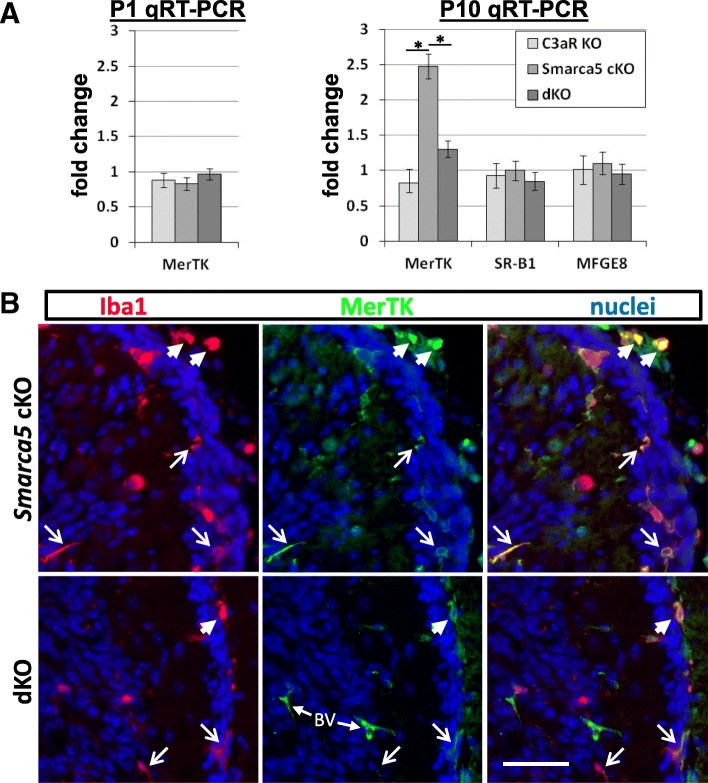
Increased MerTK expression in the *Smarca5* cKO cerebellum is attenuated in the absence of C3aR. **a** qRT-PCR analysis indicated that MerTK was increased over 2-fold in the *Smarca5* cKO cerebellum at P10 (*n* = 5 mice/genotype), but not at P1 (*n* = 3 mice/genotype). This increase was almost completely attenuated with the loss of C3aR in the dKO mice (*p < 0.05). Transcripts for two other proteins involved in efferocytosis, SR-B1 and MFG-E8 (*n* = 5 mice/genotype), were not increased in any of the mice. **b** MerTK expression was observed almost exclusively on Iba1^+^ macrophages (arrowheads) and microglia (open arrows) in the mutant cerebellums. Most other labeling with the MerTK antibody was non-specific blood vessel (BV) labeling. Scale bar = 50 μm, and applies to all panels

To examine whether the loss of C3aR altered the phenotype of phagocyte cells in the mutant cerebellums, we performed a qRT-PCR analysis of transcripts for key proteins involved in the phagocytosis of apoptotic cells (efferocytosis). We analyzed transcript expression of MerTK and SR-B1, two phagocyte receptors, and MFG-E8, which is produced by both phagocytes and Bergmann glia and functions as an opsonin during the process of efferocytosis. Of these, only MerTK showed a significant increase in expression in the *Smarca5* mutant cerebellum, with an increase of ~ 2.5-fold at P10 (Fig. 8a). Interestingly, the increase in MerTK transcript expression was virtually abolished in dKO mice. At P1, no increase was observed in any of the cerebellums analyzed relative to control mice. Thus, the increase in MerTK transcript expression occurs within the *Smarca5* cKO cerebellum only when C3aR is expressed.

In tissue sections, MerTK immunolabeling in the cerebellum was limited to Iba1^+^ phagocyte cells. MerTK showed the variable expression on Iba1^+^ cells in each of the genotypes examined (Fig. 8b). In the *Smarca5* cKO cerebellum, MerTK was present on phagocytes invading the EGL, with some displaying strong labeling and others having undetectable MerTK labeling. BAMs on the outside of the EGL were consistently observed to have stronger labeling of MerTK (Fig. 8b). This labeling was decreased in the dKO Iba1^+^ cells in and adjacent to the EGL.

## Discussion

In this study, we have demonstrated that, following neurodevelopmental damage, C3aR functions to limit the numbers of dead cerebellar granule cells present, reduces Bergmann glia disorganization, and regulates the expression of a receptor important for the clearance of dead cells. C3aR itself was only observed to be expressed by phagocyte cells in the cerebellum by immunofluorescence analysis. Phagocyte infiltration into the affected EGL region of the developing cerebellum was altered in the mutant mice, with the loss of C3aR resulting in an increased infiltration soon after birth. The overall effect of removing C3aR was to promote increased cerebellar disorganization and an exaggerated gliosis phenotype in mutants burdened with clearing apoptosing neurons.

Removal of the Snf2h protein from the nervous system results in impaired chromatin remodeling functions which are required for the successful proliferation of subsets of neuronal cells [3]. Snf2h is a subunit of the ACF/CHRAC, WICH, and NoRC remodeling complexes, wherein it functions as a motor to promote DNA replication, repair, and transcription [3, 23]. The defective replication of heterochromatin can result in DNA damage, mitotic catastrophe and cell death [24–27]. The absence of Snf2h in blood cells has also been reported to induce p53 activation, leading to apoptotic death [23]. In the cerebellum, a massive expansion of granule neuron precursors is critical to establish proper foliation [28], and death of these cells in the *Smarca5* cKO brain is likely the primary cause of their small, disorganized cerebellum.

The C3- and VGF-derived C3aR agonists, C3a and TLQP-21 (a segment of TLQP-62), have both been described as affecting granule neurons during cerebellar development, though in different ways [29–31]. Despite the fact that we did not observe C3aR expression on cells other than Iba1^+^ phagocytes, these prior studies have indicated that C3aR is at least transiently expressed by granule neurons. Our lack of detection of C3aR on granule cells by immunofluorescence analysis may indicate low levels of neuronal expression relative to its expression on phagocytes. We note here that other recent studies have also indicated that C3aR expression in the brain occurs almost exclusively on phagocyte cells, with uniform expression on brain macrophages and selective expression on microglial subpopulations [32–34]. Nonetheless, functional data has demonstrated that C3a promotes the migration of granule neurons from the EGL to the IGL [30]. TLQP-21, on the other hand, was demonstrated to protect granule neurons from serum and potassium deprivation-induced death in vitro [31]. These studies indicate that C3aR signaling may impact directly on granule neurons, at least during a transient period of cerebellar development. Direct signaling through C3aR on granule neurons in the nascent cerebellum may account for the increased numbers of apoptotic cells found at P1 in the dKO cerebellum (Fig. 6).

Prior evidence has demonstrated that the death of granule neurons in the developing cerebellum can result in an inflamed phenotype within the Bergmann glia [35]. Bergmann glial inflammation coincident with phagocyte invasion into the EGL following granule neuron apoptosis occurs in the rat cerebellum following treatment with the cytotoxic agent, methylazoxymethanol [35]. We speculate that within the *Smarca5* cKO mice, the presence of apoptotic bodies early on during cerebellum development triggers a similar, though much stronger gliosis phenotype. The granule neuron loss in the *Smarca5* cKO mice occurs early and in large numbers, resulting in an increase of GFAP protein expression that is noticeable at P10 (Fig. 5) and 8–9 fold higher by P35 (Fig. 1; Additional file 3).

The predominant expression of C3aR on microglia and macrophage cells indicates that the exacerbated Bergmann glia disorganization in the *Smarca5;C3aR* dKO mice likely occurs, at least in part, as a result of impaired functioning of these cells. An inability to upregulate MerTK expression in the dKO cerebellum by P10 is consistent with an impaired ability of the phagocytes to clear dead cells. This may account for the higher numbers of TUNEL^+^ cells in the dKO EGL (Fig. 6). The presence of dead cells can act as at least one trigger driving the exacerbated Bergmann glia inflammation, as the defective clearance of apoptotic cells results in a conversion to necrotic death and the release of danger associated molecular patterns (DAMPS) and inflammatory cytokines [36].

Activation of the C1q complement protein is well known to be important for promoting the clearance of apoptotic cells, acting to do so through several different mechanisms [36, 37]. One of these mechanisms is through the promotion of MerTK expression by macrophages [38]. C1q, in general, directs macrophages to adopt a pro-efferocytic phenotype and to limit inflammation, in part by limiting the production of the pro-inflammatory cytokine, TNFα [39]. While C1q can promote an efferocytic phenotype in vitro, some evidence has indicated that C3 activation is required downstream of C1q activation for the proper clearance of apoptotic cells in vivo [36]. The cleavage product of C3, C3b, functions as an opsonin, and can do so in bridging apoptotic cells and phagocytes to promote efferocytosis [40]. Autoantibodies that prevent the deposition of C3b can inhibit the clearance of apoptotic cells and exacerbate autoimmunity [41]. However, to our knowledge, a role for C3a in this process has not yet been described.

We speculate that C3- and VGF-derived peptides may play a role in this process in young mice experiencing developmental cell loss, though further examination is needed to determine the details of this involvement. In older *Smarca5* cKO mutant mice (P35), in which both C3 and VGF transcripts were found to have been upregulated, active peptides from these proteins may continue to play a role in clearing dead cell debris or may play additional roles. Indeed, C-terminal VGF peptides have also been implicated in, as stated above, promoting neuronal survival, as well as in promoting myelination in the brain.

## Conclusions

The phenotype observed with the loss of C3aR function in this study points to a reparative role for this receptor, and for complement signaling in general, following neurodevelopmental damage. The altered processing of C3 in the *Smarca5* cKO cerebellum indicates its activation in this model, along with the activation of a second C3aR signaling protein, VGF. Loss of C3aR resulted in an exaggerated gliosis phenotype and dysregulation of a key efferocytosis-related protein, MerTK. Together, this indicates that C3aR and its agonists may be required for promoting the efficient clearance of apoptotic cells during abnormal development, thereby preventing the accumulation of necrotic cells and the promotion of excessive inflammation.

## Additional files

Additional file 1: SNP analysis of different mouse lines used in this study. (DOCX 14 kb)

Additional file 2: Primer sequences used for quantitative RT-PCR. (DOCX 14 kb)

Additional file 3: Normalized GFAP expression in Snf2h cKO mice. Quantification of GFAP expression of immunoblot in Fig. 1c. Expression of GFAP was first normalized to the actin loading control and then to WT sedentary mice. Running induced a 1.2-fold increase in GFAP levels in WT mice. However, the removal of Snf2h resulted in an 8.4-fold increase in GFAP expression that was slightly attenuated (6.0-fold increase) when a running wheel was provided. (DOCX 64 kb)

Additional file 4: Expression of C3aR and gC1qR on microglia and macrophages. (A) Labeling for both receptors was observed on macrophages (arrows) in the subarachnoid space of the cerebellum. The expression of each was heterogenous, with different macrophages labeling more strongly for one or the other. (B) In *Smarca5* mutants bred on a C57BL/6 background, C3aR labeling was more consistently labeled on microglial cells (thin open arrows) in addition to the macrophages (thicker arrows), though this microglial labeling was also consistently weaker than the macrophage labeling. Scale bar = 50 μm, and applies to all images. (DOCX 120 kb)

Additional file 5: Growth curves of *Smarca5* cKO, *C3aR* KO, and dKO mice in comparison to control littermates. Number of animals in each group is shown on the right of each graph; error bars represent the SEM. (DOCX 80 kb)

Additional file 6: Bergmann glia and Purkinje cell disorganization in different lobes of the P10 *Smarca5*; *C3aR* dKO and control cerebellum. The higher magnification images are from reconstructed optical sections. Scale bar = 100 μm in lower magnification image; 50 μm in higher magnification image. (DOCX 122 kb)

Additional file 7: VGF labeling in the P10 *Smarca5*;*C3aR* dKO cerebellum. Arrows point to VGF labeling within calbindin-labeled Purkinje cell dendrites that are closely apposed to BLBP-labeled Bergmann glia processes. The images are from reconstructed optical sections. Scale bar = 50 μm, and applies to all images. (DOCX 129 kb)

Additional file 8: Endothelial protein expression indicates no abnormalities in the blood-brain-barrier (BBB) of P10 *Smarca5* cKO and dKO mutants. Labeling of both the claudin-5 (A) and ZO-1 (B) tight junction proteins was similar in the P10 cerebellum of control and mutant mice. PLVAP is a protein that is downregulated in the brain following acquisition of intact BBB properties during development. PLVAP labeling was absent from blood vessels in the cerebellum of all genotypes. In the choroid plexus (C), which contains fenestrated blood vessels that are leaky, PLVAP expression was present in all genotypes, as expected. Antibodies used for this labeling were: rabbit anti-mouse claudin-5 (ThermoFisher); mouse anti-ZO-1, clone 1A12 (ThermoFisher); and rat anti-mouse PLVAP, clone MECA-32 (BD Biosciences). (DOCX 367 kb)

Additional file 9: Influx of Iba1^+^ cells in the mutant EGL that do not express the P2RY12 microglial marker. Representative images of the EGL region from the cerebellum of WT, C3aR KO, *Smarca5* cKO, or dKO mice (*n*=3) stained with Iba1 (green) and P2RY12 (red) antibodies. Arrows highlight the Iba1+ cells that were not co-labeled with P2RY12 and are suggestive of invading macrophages. Scale bar = 20 μm. (DOCX 1546 kb)

## Data Availability

The full data set for the RNAseq data presented in Fig. 1 can be found at https://www.ncbi.nlm.nih.gov/geo/query/acc.cgi?acc=GSE86235. The *C3aR1* KO mutant line used in this study is *C3ar1*^*tm1Cge*^/J, available from The Jackson Laboratory. *Smarca5* mutant mice carrying either floxed or null alleles were originally obtained from Dr. Arthur Skoultchi at the Albert Einstein College of Medicine. *VGF* floxed mice were obtained from Dr. Stephen Salton, Icahn School of Medicine, Mount Sinai, New York.

## Abbreviations

BLBP: Brain lipid binding protein
CNS: Central nervous system
EGL: External granule cell layer
GFAP: Glial fibrillary acidic protein
IGL: Inner granule cell layer
MerTK: Mer tyrosine kinase
Snf2h: Sucrose nonfermenting protein 2 homolog, also SWI/SNF-related matrix-associated actin-dependent regulator of chromatin A5
SRB1: Scavenger receptor-B1
