# Correction to: C3aR signaling and gliosis in response to neurodevelopmental damage in the cerebellum

**DOI:** 10.1186/s12974-019-1642-x

**Published:** 2020-01-23

**Authors:** Kevin G. Young, Keqin Yan, David J. Picketts

**Affiliations:** 1 Regenerative Medicine Program, Ottawa Hospital Research Institute, Ottawa, ON K1H 8L6 Canada; 2 Present Address: Therapeutic Products Directorate, Health Canada, 1600 Scott St, Ottawa, ON K1A 0K9 Canada; 3 Department of Cellular and Molecular Medicine, University of Ottawa, Ottawa, ON K1H 8M5 Canada; 4 Department of Biochemistry, Microbiology, and Immunology, University of Ottawa, Ottawa, ON K1H 8M5 Canada

**Correction to: J Neuroinflammation**

**https://doi.org/10.1186/s12974-019-1530-4**

Following publication of the original article [1], the authors noticed missing labels in Fig. 1a. The bar graph contains the labels C3, C2, C1ql2, C1ql1, C1qb, GFAP, and VGF. However, the labels should be C3, C4b, C2, C3aR1, C1ql2, C1ql4, C1ql1, C1qa, C1qb, C1qc, GFAP, USP18, and VGF. The correct version of Fig. 1a is published in this Erratum.

**Fig. 1:**
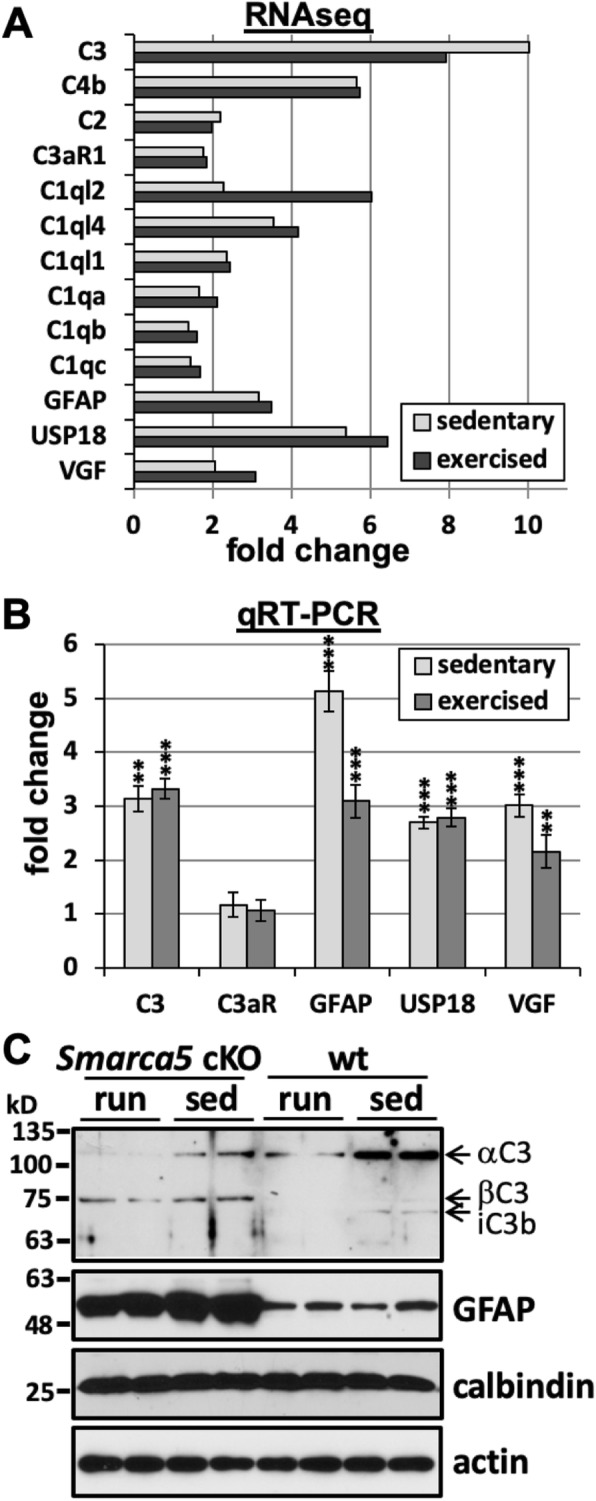
Altered C3 complement protein expression in the *Smarca5* cKO cerebellum of exercised and sedentary mice. Increases in mRNA transcripts coding for complement, complement-related proteins, and inflammation-related proteins in the *Smarca5* cKO cerebellum, as indicated by RNAseq analysis (**a**). Fold changes are shown for the *Smarca5* cKO groups (sedentary or exercised) relative to corresponding wild-type groups. qRT-PCR analysis confirmed the increases in C3, GFAP, USP18, and VGF (**b**), though the magnitudes of these increases varied from the RNAseq data set. Shown are the fold changes in the *Smarca5* cKO cerebellum relative to wild-type littermates (*n* = 3 in each of the four groups of wild-type exercised (run) or sedentary (sed), and mutant exercised or sedentary animals; differences relative to wild-type littermates are noted with **p < 0.005 and ***p < 0.001). No increase was observed for the C3a receptor, C3aR. Protein analysis demonstrated a clear increase in GFAP expression in *Smarca5* cKO cerebellum samples (**c**). C3 protein expression was also altered in the *Smarca5* cKO cerebellum. The C3α chain was less prominent relative to the C3β chain in *Smarca5* cKO cerebellum samples compared to wild-type samples. Blotting results are representative of similar results from four mice/group
