# Supplementary material for: C3aR signaling and gliosis in response to neurodevelopmental damage in the cerebellum
Source: J Neuroinflammation. 2019 Jul 4;16:135. doi: 10.1186/s12974-019-1530-4 (PMC6610970; doi:10.1186/s12974-019-1530-4)
Supplement: Supplementary file 1 — SNP analysis of different mouse lines used in this study. (DOCX 14 kb) [file 12974_2019_1530_MOESM1_ESM.docx]

| **Animal ID** | **Sex** | **% FVB/N^*^** | **% C57BL/6N^*^** | **% BALB/c^*^** | **Genotype** |
| --- | --- | --- | --- | --- | --- |
| **1** | **M** | **50.42%** | **90.55%** | **48.02%** | **Snf2H f/f (C57/BL6)** |
| **2** | **F** | **80.12%** | **62.44%** | **55.21%** | **Snf2h f/f (FVB/N)** |
| **3** | **M** | **69.82%** | **51.39%** | **82.10%** | **Snf2H+/-; C3aR-/-; Nestin cre +/-** |
| **4** | **F** | **70.60%** | **50.81%** | **81.37%** | **Snf2H f/f; C3aR-/-** |

*, percentage of shared SNPs (2050 tested) to reference strain.

Additional File 1: **Table S1.** SNP analysis of different mouse lines used in this study.
