# Supplementary material for: C3aR signaling and gliosis in response to neurodevelopmental damage in the cerebellum
Source: J Neuroinflammation. 2019 Jul 4;16:135. doi: 10.1186/s12974-019-1530-4 (PMC6610970; doi:10.1186/s12974-019-1530-4)
Supplement: Supplementary file 2 — Primer sequences used for quantitative RT-PCR. (DOCX 14 kb) [file 12974_2019_1530_MOESM2_ESM.docx]

|  | **forward** | **reverse** |
| --- | --- | --- |
| **GFAP** | 5'-CAACCTGGCTGCGTATAGACAGGA-3' | 5'-GAATCTCTCTCAGGGCCGCTGTG-3' |
| **C3** | 5'-ATTAGCTCTGGGGATCCCCATGT-3' | 5'-CTTGATGGAGACGCTTCTCAGATG-3' |
| **C3aR** | 5'-CAGGGAAAAGTCAGTGCTCAG-3' | 5'-GGCCGTGAGTGTAGGTCAGT-3' |
| **VGF** | 5'-TCGCTCATACTCCAGCCACG-3' | 5'-TCCCAACCCCTGGATCAGTAG-3' |
| **USP18** | 5'-ACAGCCCTCATGGTCTGGTT-3' | 5'-CGGCTTTCTGTCGACTGTCC-3' |
| **Iba1** | 5'-AAAGCAGGGATTTGCAGGGAG-3' | 5'-TTCTCCAGCATTCGCTTCAAGG-3' |
| **gC1qR** | 5'-AACGGCACGGAGGCTAAAT-3' | 5'-AAGGGTCTTCTTGCCATCAGT-3' |
| **IL6** | 5'-TCCGGAGAGGAGACTTCACA-3' | 5'-TCCAGTTTGGTAGCATCCATCA-3' |
| **TNF** | 5'-GTGACAAGCCTGTAGCCCAC-3' | 5'-GATAGCAAATCGGCTGACGG-3' |
| **MerTK** | 5'-ATCTCACGTGTGGGAAAGCG-3' | 5'-GACGAGGGTGCGTAATCTACC-3' |
| **SR-B1** | 5'-GCCTCTGTTTCTCTCCCACC-3' | 5'-CTGTTCGAACCACAGCAACG-3' |
| **MFGE8** | 5'-TGGACAGCCAGCAACTATGA-3' | 5'-CTCCTTGTCTCCACCGCTTT-3' |

Additional file 2: **Table S2** Primer sequences used for quantitative RT-PCR.
