# Supplementary material for: C3aR signaling and gliosis in response to neurodevelopmental damage in the cerebellum
Source: J Neuroinflammation. 2019 Jul 4;16:135. doi: 10.1186/s12974-019-1530-4 (PMC6610970; doi:10.1186/s12974-019-1530-4)
Supplement: Supplementary file 3 — Normalized GFAP expression in Snf2h cKO mice. Quantification of GFAP expression of immunoblot in Fig. 1c. Expression of GFAP was first normalized to the actin loading control and then to WT sedentary mice. Running induced a 1.2-fold increase in GFAP levels in WT mice. However, the removal of Snf2h resulted in an 8.4-fold increase in GFAP expression that was slightly attenuated (6.0-fold increase) when a running wheel was provided. (DOCX 64 kb) [file 12974_2019_1530_MOESM3_ESM.docx]

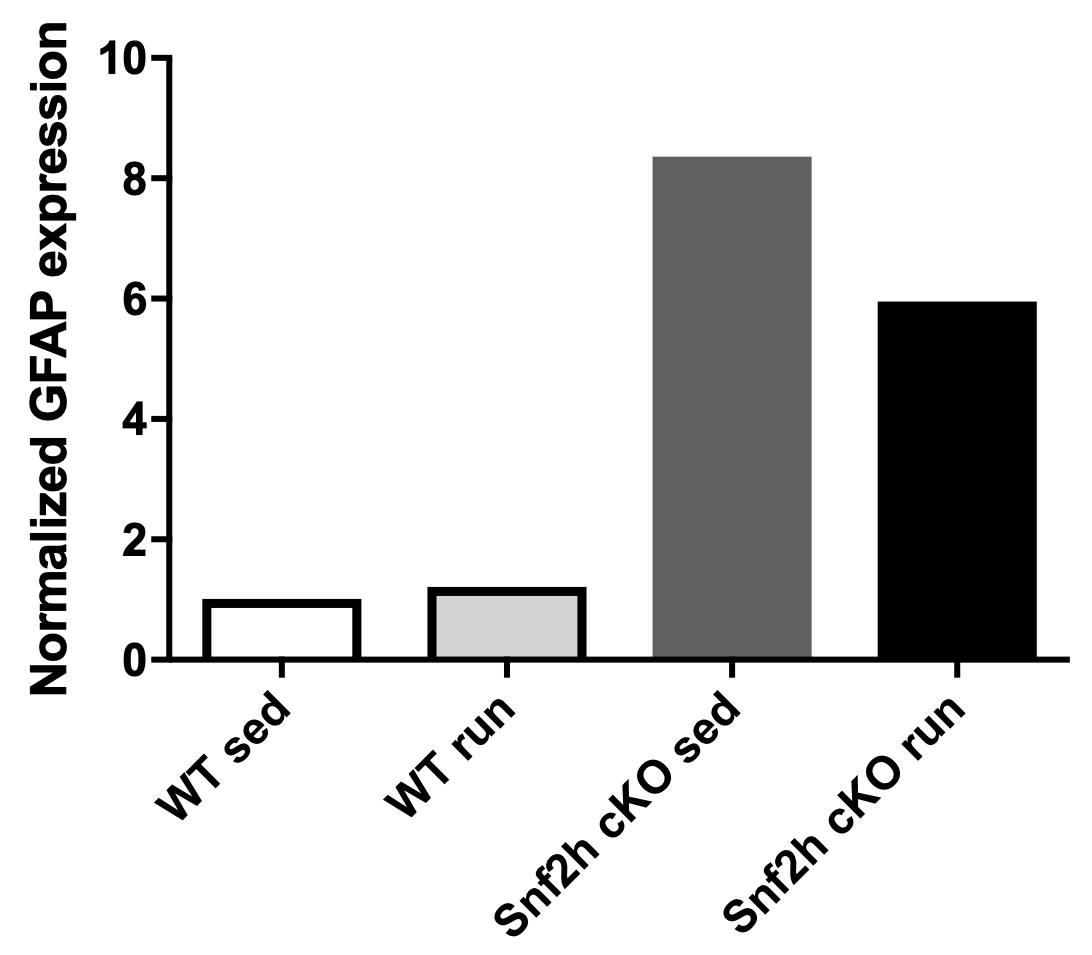


Additional file 3: **Figure S1** Normalized GFAP expression in Snf2h cKO mice. Quantification of GFAP expression of immunoblot in Figure 1C. Expression of GFAP was first normalized to the actin loading control and then to WT sedentary mice. Running induced a 1.2-fold increase in GFAP levels in WT mice. However, the removal of Snf2h resulted in an 8.4-fold increase in GFAP expression that was slightly attenuated (6.0-fold increase) when a running wheel was provided.
