# Supplementary material for: C3aR signaling and gliosis in response to neurodevelopmental damage in the cerebellum
Source: J Neuroinflammation. 2019 Jul 4;16:135. doi: 10.1186/s12974-019-1530-4 (PMC6610970; doi:10.1186/s12974-019-1530-4)
Supplement: Supplementary file 4 — Expression of C3aR and gC1qR on microglia and macrophages. (A) Labeling for both receptors was observed on macrophages (arrows) in the subarachnoid space of the cerebellum. The expression of each was heterogenous, with different macrophages labeling more strongly for one or the other. (B) In Smarca5 mutants bred on a C57BL/6 background, C3aR labeling was more consistently labeled on microglial cells (thin open arrows) in addition to the macrophages (thicker arrows), though this microglial labeling was also consistently weaker than the macrophage labeling. Scale bar = 50 μm, and applies to all images. (DOCX 120 kb) [file 12974_2019_1530_MOESM4_ESM.docx]

**
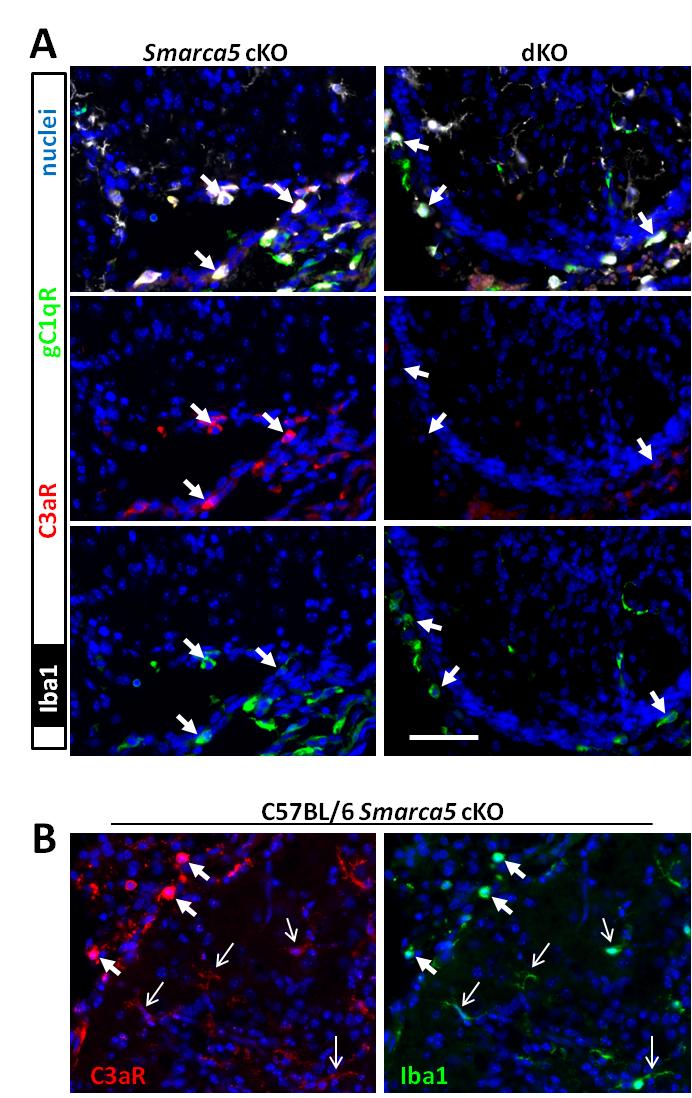
**

Additional file 4: **Figure S2** Expression of C3aR and gC1qR on microglia and macrophages. (**A**) Labeling for both receptors was observed on macrophages (arrows) in the subarachnoid space of the cerebellum. The expression of each was heterogenous, with different macrophages labeling more strongly for one or the other. (**B**) In *Smarca5* mutants bred on a C57BL/6 background, C3aR labeling was more consistently labeled on microglial cells (thin open arrows) in addition to the macrophages (thicker arrows), though this microglial labeling was also consistently weaker than the macrophage labeling. Scale bar = 50 µm, and applies to all images.
