# Supplementary material for: C3aR signaling and gliosis in response to neurodevelopmental damage in the cerebellum
Source: J Neuroinflammation. 2019 Jul 4;16:135. doi: 10.1186/s12974-019-1530-4 (PMC6610970; doi:10.1186/s12974-019-1530-4)
Supplement: Supplementary file 5 — Growth curves of Smarca5 cKO, C3aR KO, and dKO mice in comparison to control littermates. Number of animals in each group is shown on the right of each graph; error bars represent the SEM. (DOCX 80 kb) [file 12974_2019_1530_MOESM5_ESM.docx]

**
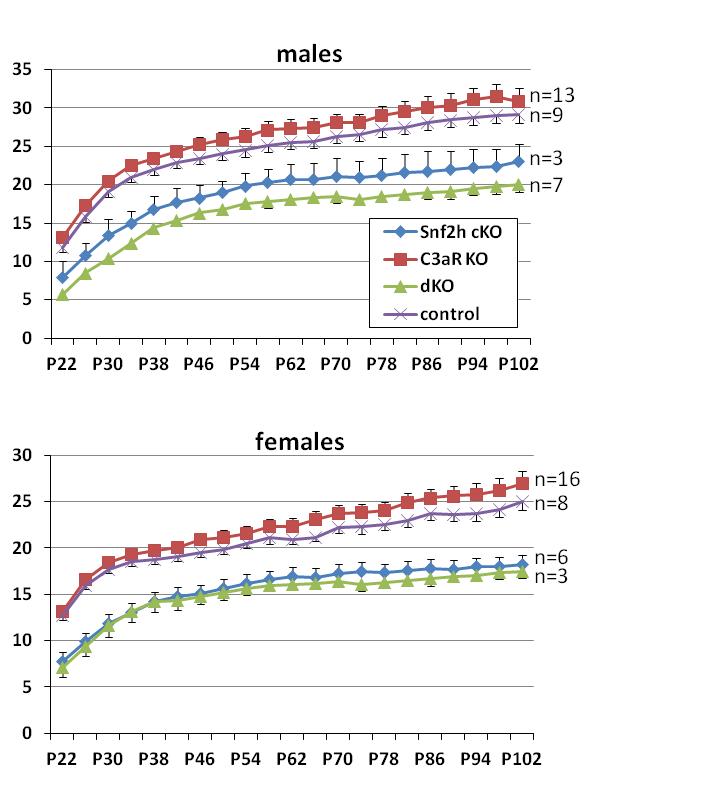
**

Additional file 5: **Figure S3** Growth curves of *Smarca5* cKO, *C3aR* KO, and dKO mice in comparison to control littermates. Number of animals in each group is shown on the right of each graph; error bars represent the SEM.
