# Supplementary material for: C3aR signaling and gliosis in response to neurodevelopmental damage in the cerebellum
Source: J Neuroinflammation. 2019 Jul 4;16:135. doi: 10.1186/s12974-019-1530-4 (PMC6610970; doi:10.1186/s12974-019-1530-4)
Supplement: Supplementary file 6 — Bergmann glia and Purkinje cell disorganization in different lobes of the P10 Smarca5; C3aR dKO and control cerebellum. The higher magnification images are from reconstructed optical sections. Scale bar = 100 μm in lower magnification image; 50 μm in higher magnification image. (DOCX 122 kb) [file 12974_2019_1530_MOESM6_ESM.docx]

**
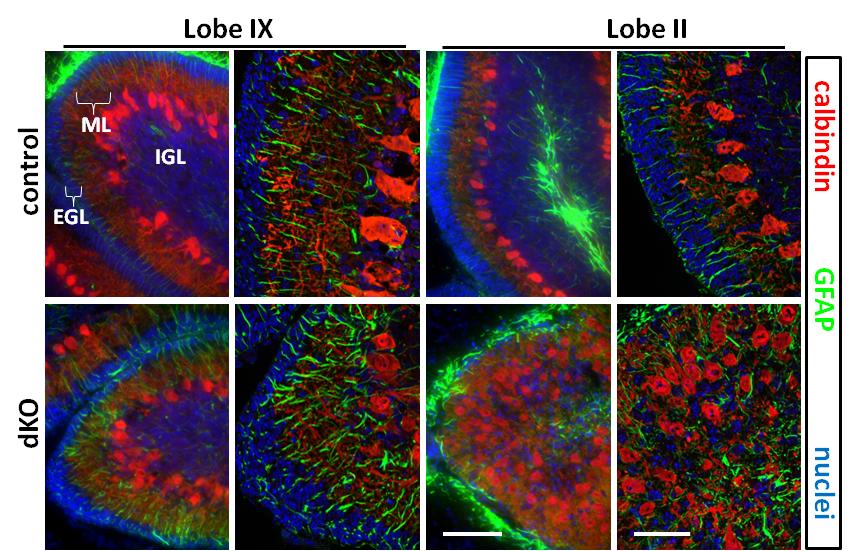
**

Additional file 6: **Figure S4** Bergmann glia and Purkinje cell disorganization in different lobes of the P10 *Smarca5*; *C3aR* dKO and control cerebellum. The higher magnification images are from reconstructed optical sections. Scale bar = 100 µm in lower magnification image; 50 µm in higher magnification image.
