# Supplementary material for: C3aR signaling and gliosis in response to neurodevelopmental damage in the cerebellum
Source: J Neuroinflammation. 2019 Jul 4;16:135. doi: 10.1186/s12974-019-1530-4 (PMC6610970; doi:10.1186/s12974-019-1530-4)
Supplement: Supplementary file 7 — VGF labeling in the P10 Smarca5;C3aR dKO cerebellum. Arrows point to VGF labeling within calbindin-labeled Purkinje cell dendrites that are closely apposed to BLBP-labeled Bergmann glia processes. The images are from reconstructed optical sections. Scale bar = 50 μm, and applies to all images. (DOCX 129 kb) [file 12974_2019_1530_MOESM7_ESM.docx]

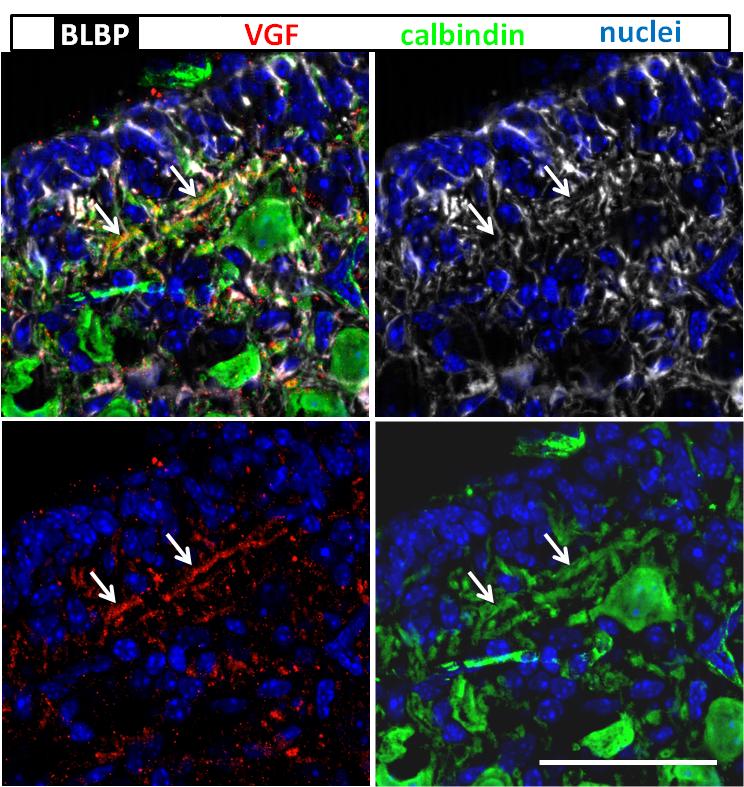


Additional file 7: **Figure S5.** VGF labeling in the P10 *Smarca5*;*C3aR* dKO cerebellum. Arrows point to VGF labeling within calbindin-labeled Purkinje cell dendrites that are closely apposed to BLBP-labeled Bergmann glia processes. The images are from reconstructed optical sections. Scale bar = 50 µm, and applies to all images.
