# Supplementary material for: C3aR signaling and gliosis in response to neurodevelopmental damage in the cerebellum
Source: J Neuroinflammation. 2019 Jul 4;16:135. doi: 10.1186/s12974-019-1530-4 (PMC6610970; doi:10.1186/s12974-019-1530-4)
Supplement: Supplementary file 8 — Endothelial protein expression indicates no abnormalities in the blood-brain-barrier (BBB) of P10 Smarca5 cKO and dKO mutants. Labeling of both the claudin-5 (A) and ZO-1 (B) tight junction proteins was similar in the P10 cerebellum of control and mutant mice. PLVAP is a protein that is downregulated in the brain following acquisition of intact BBB properties during development. PLVAP labeling was absent from blood vessels in the cerebellum of all genotypes. In the choroid plexus (C), which contains fenestrated blood vessels that are leaky, PLVAP expression was present in all genotypes, as expected. Antibodies used for this labeling were: rabbit anti-mouse claudin-5 (ThermoFisher); mouse anti-ZO-1, clone 1A12 (ThermoFisher); and rat anti-mouse PLVAP, clone MECA-32 (BD Biosciences). (DOCX 367 kb) [file 12974_2019_1530_MOESM8_ESM.docx]

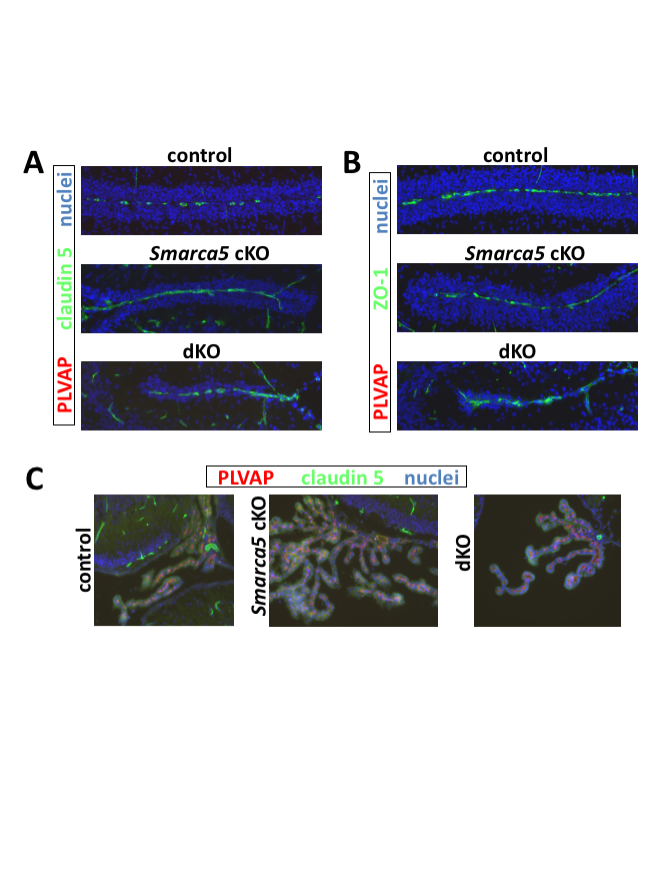


Additional File 8: **Figure S6** Endothelial protein expression indicates no abnormalities in the blood-brain-barrier (BBB) of P10 *Smarca5* cKO and dKO mutants. Labeling of both the claudin-5 (**A**) and ZO-1 (**B**) tight junction proteins was similar in the P10 cerebellum of control and mutant mice. PLVAP is a protein that is down-regulated in the brain following acquisition of intact BBB properties during development. PLVAP labeling was absent from blood vessels in the cerebellum of all genotypes. In the choroid plexus (**C**), which contains fenestrated blood vessels that are leaky, PLVAP expression was present in all genotypes, as expected. Antibodies used for this labeling were: rabbit anti-mouse claudin-5 (ThermoFisher); mouse anti-ZO-1, clone 1A12 (ThermoFisher); and rat anti-mouse PLVAP, clone MECA-32 (BD Biosciences).
