# Supplementary material for: C3aR signaling and gliosis in response to neurodevelopmental damage in the cerebellum
Source: J Neuroinflammation. 2019 Jul 4;16:135. doi: 10.1186/s12974-019-1530-4 (PMC6610970; doi:10.1186/s12974-019-1530-4)
Supplement: Supplementary file 9 — Influx of Iba1+ cells in the mutant EGL that do not express the P2RY12 microglial marker. Representative images of the EGL region from the cerebellum of WT, C3aR KO, Smarca5 cKO, or dKO mice (n=3) stained with Iba1 (green) and P2RY12 (red) antibodies. Arrows highlight the Iba1+ cells that were not co-labeled with P2RY12 and are suggestive of invading macrophages. Scale bar = 20 μm. (DOCX 1546 kb) [file 12974_2019_1530_MOESM9_ESM.docx]

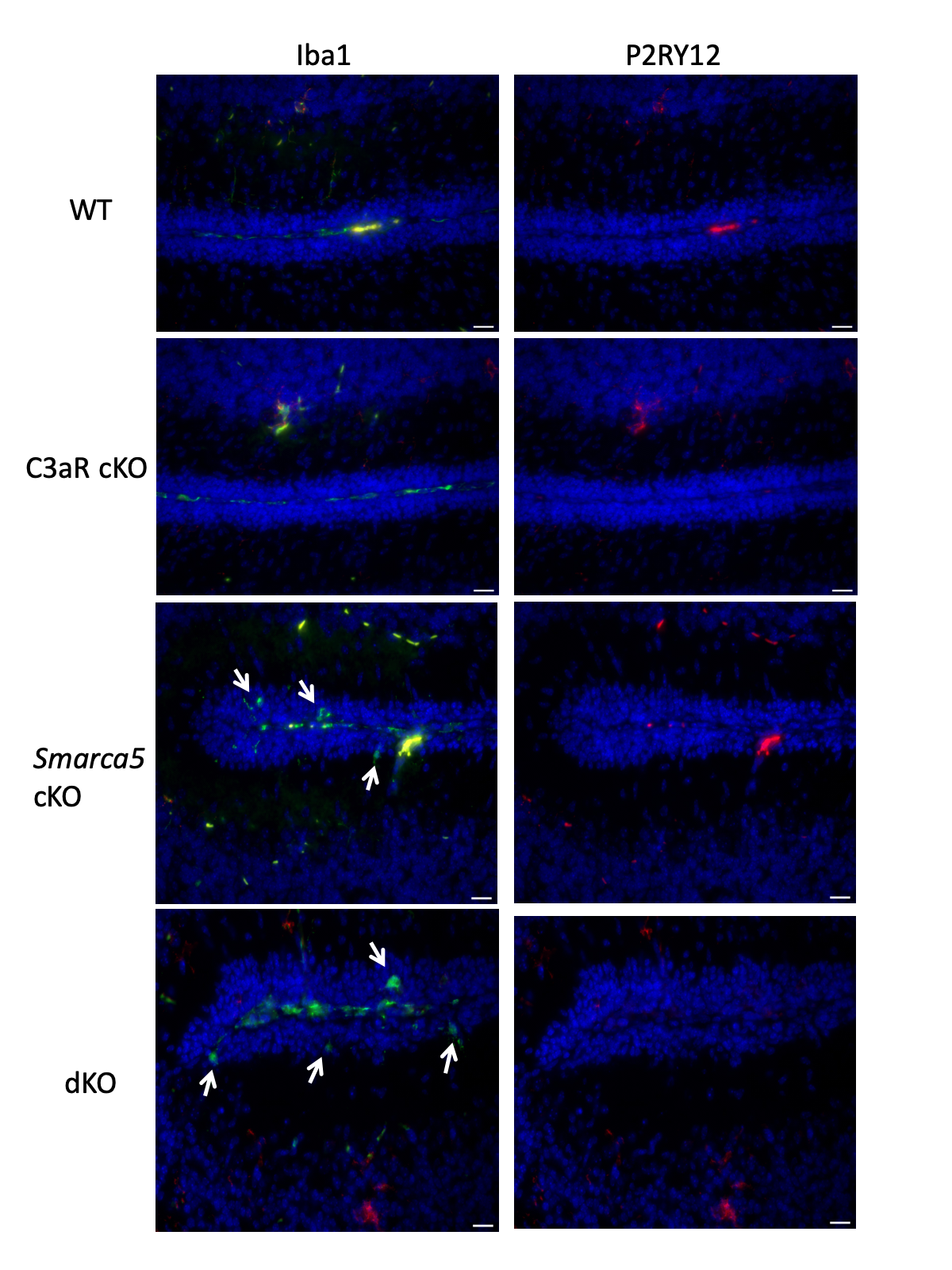


Additional File 9: **Figure S7.** Influx of Iba1^+^ cells in the mutant EGL that do not express the P2RY12 microglial marker. Representative images of the EGL region from the cerebellum of WT, C3aR KO, *Smarca5* cKO, or dKO mice (n=3) stained with Iba1 (green) and P2RY12 (red) antibodies. Arrows highlight the Iba1+ cells that were not co-labeled with P2RY12 and are suggestive of invading macrophages. Scale bar = 20 µm.
